# The Genetics of Primary Haemorrhagic Stroke, Subarachnoid Haemorrhage and Ruptured Intracranial Aneurysms in Adults

**DOI:** 10.1371/journal.pone.0003691

**Published:** 2008-11-14

**Authors:** George Peck, Liam Smeeth, John Whittaker, Juan Pablo Casas, Aroon Hingorani, Pankaj Sharma

**Affiliations:** 1 Imperial College Cerebrovascular Research Unit (ICCRU), Department of Clinical Neuroscience, Imperial College London & Hammersmith Hospitals, London, United Kingdom; 2 Department of Epidemiology and Population Health, London School of Hygiene & Tropical Medicine, University of London, London, United Kingdom; 3 Centre for Clinical Pharmacology, BHF Laboratories, University of London, London, United Kingdom; Western General Hospital, United Kingdom

## Abstract

**Background:**

The genetic basis of haemorrhagic stroke has proved difficult to unravel, partly hampered by the small numbers of subjects in any single study. A meta-analysis of all candidate gene association studies of haemorrhagic stroke (including ruptured subarachnoid haemorrhage and amyloid angiopathy-related haemorrhage) was performed, allowing more reliable estimates of risk.

**Methods:**

A systematic review and meta-analysis of all genetic studies in haemorrhagic stroke was conducted. Electronic databases were searched until and including March 2007 for any candidate gene in haemorrhagic stroke. Odds ratio (OR) and 95% confidence intervals (CI) were determined for each gene disease association using fixed and random effect models.

**Results:**

Our meta-analyses included 6,359 cases and 13,805 controls derived from 55 case-control studies, which included 12 genes (13 polymorphisms). Statistically significant associations with haemorrhagic stroke were identified for those homozygous for the ACE/I allele (OR, 1.48; 95% CI, 1.20–1.83; p = 0.0003) and for the 5G allele in the SERPINE1 4G/5G polymorphism (OR, 1.42; 95% CI, 1.03–1.96; *p* = 0.03). In addition, both &b.epsi;2 and &b.epsi;4 alleles of APOE were significantly associated with lobar haemorrhage (OR, 1.81; 95% CI, 1.26–2.62; *p* = 0.002 and OR, 1.49; 95% 1.08–2.05; *p* = 0.01 respectively). Furthermore, a significant protective association against haemorrhagic stroke was found for the factor V Leiden mutation (OR, 0.30; 95% CI, 0.10–0.87; p = 0.03).

**Conclusion:**

Our data suggests a genetic contribution to some types of haemorrhagic stroke, with no overall responsible single gene but rather supporting a polygenic aetiology . However, the evidence base is smaller compared to ischaemic stroke. Importantly, for several alleles previously found to be associated with protection from ischaemic stroke, there was a trend towards an increased risk of haemorrhagic stroke.

## Introduction

Haemorrhagic stroke accounts for approximately 13% of all stroke and is associated with a mortality rate four times higher than ischaemic stroke,[1], [2] with only 38% of haemorrhagic stroke patients surviving beyond the first year.[3] Intracranial haemorrhage can be subdivided into extradural, subdural, subarachnoid, intraventricular and intracerebral (with/without subarachnoid and/or intraventricular) haemorrhage. Primary intracerebral haemorrhage (ICH) originates from the spontaneous rupture of small vessels damaged by chronic hypertension or cerebral amyloid angiopathy,[4] while secondary intracerebral haemorrhage is most commonly associated with subarachnoid haemorrhage (SAH)_due to ruptured intracranial aneurysms or ruptured arteriovenous malformations. Haemorrhage from rupture of hypervascular tumors or impaired coagulation occurs in a very small proportion of cases while traumatic related haemorrhage tends to have different characteristics.

Although significant progress has been made towards unraveling the basis of single gene stroke disorders and common ischaemic stroke [5], [6], identifying the underlying genes for multifactorial haemorrhagic stroke for which there is no obvious Mendelian pattern of inheritance, has proved difficult. This is despite the evidence for a genetic contribution towards haemorrhagic stroke being considerable (with a likely greater genetic effect for SAH compared to ICH). Stroke cases have also been found to cluster in families. Approximately 10% of patients with intracerebral haemorrhage have a positive family history of haemorrhagic stroke [7], and having a first-degree relative with intracerebral haemorrhage has been found to be a risk factor for developing the disease. First degree relatives of patients with subarachnoid haemorrhage have up to a seven-fold increased risk of subarachnoid haemorrhage than the general population,[8], [9] and about 10% of patients with aneurysmal subarachnoid haemorrhage have a first or second degree relative with subarachnoid haemorrhage or unruptured intracranial aneurysm.[10], [11]

Genetic association studies in haemorrhagic stroke have identified a considerable number of candidate genes, however, due to lack of reproducibility, uncertainty remains about the nature and number of genes contributing to risk of haemorrhagic stroke.[6] There is concern, on one hand, that positive associations might be spurious and, on the other hand, that the negative findings from some studies might be a consequence of inadequate statistical power from small sample size or methodological shortcomings, such as selection of inappropriate control groups.[12], [13] The use of a genetic meta-analysis strategy overcomes some of these limitations, although the results are limited by the available data. Therefore, in this study a comprehensive literature-based meta-analysis of all published association studies in mainly primary intracerebral and aneurysmal subarachnoid forms of haemorrhage was undertaken.

## Methods

### Data sources

Electronic searches initially using PubMed, Embase and Google Scholar were used to identify published genetic association studies evaluating any candidate gene and any form of haemorrhagic stroke in humans published until and including March 2007. Letters and abstracts were included in the searches.

The Medical Subject Headings and text words used for the search were: *haemorrhagic stroke*, *intracerebral haemorrhage*, *intracranial haemorrhage*, *subarachnoid haemorrhage*, *intracranial aneurysm*, *aneurysmal h(a)emorrhage*, *arteriovenous malformation*, *cerebrovascular malformation*, *cavernous angioma* and *cerebral amyloid angiopathy* in combination with *polymorphism*, *gene*, *genotype* or *mutation*. Search results were limited to ‘*human*’. All languages were searched and translated when necessary. The references of all identified publications were searched for additional studies and the MEDLINE option *related articles* was used to examine all relevant articles.

### Study selection

Selection criteria included case-control or cohort studies where stroke was analyzed as a dichotomous trait. Studies were selected if neuroimaging (magnetic resonance imaging, computerised tomography or angiography) or autopsy had been used to confirm a diagnosis of intracerebral haemorrhage, subarachnoid haemorrhage, ruptured intracranial aneurysm or cerebral amyloid angiopathy-related haemorrhage. *Control populations:* The vast majority of the time subjects classified as ‘controls’ had not been subjected to neuro-imaging with investigators assuming that any haemorrhage would be clinically detectable. Different sub-types of haemorrhages were combined on the basis that its aetiology, in the broadest sense, is vessel damage. Moreover, most investigators in our study combined such data. However, haemorrhagic sub-type data was analyzed separately where possible, e.g. Factor V Leiden. Studies of all ethnic backgrounds were included. Genes with three or more publications on each SNP were included in our analyses but, if very large numbers of subjects were identified in only two studies dealing with a solitary SNP these were included. Studies were excluded if (1) the patients were children (aged <18 years), (2) genotype frequency was not adequately reported (and such data could not be obtained from the authors), (3) genotype frequency of haemorrhagic stroke sub-types were not reported independently from ischaemic stroke cases, (4) cases due to trauma, haemorrhagic rupture of tumour or haemorrhagic conversion of ischaemic stroke were included in case genotype frequencies, or (5) quantitative traits or intermediate phenotypes were investigated. Data from SNP investigated in three or more studies are presented.

### Data extraction

The first pass data extraction was undertaken by GP. Several subsequent passes were then undertaken by GP and PS to ensure comprehensive inclusion of appropriate studies. Once studies were identified data was extracted initially by GP and then checked by PS. For studies with more than one publication describing results among the same or overlapping groups of patients or controls the largest of the available published data sets was included to avoid double counting. For studies with more than one control group, the most appropriate control group was used. Where neither control group was methodologically superior the largest was used.

### Statistical analysis

Data were analyzed using software for preparing and maintaining Cochrane reviews (Review Manager v4.2.8, Cochrane Collaboration, http://www.cc-ims.net/RevMan) and Comprehensive Meta Analysis v2.2.023 (Biostat, http://www.biostat.org). To determine the strength of genetic association a pooled odds ratio (OR) was calculated for each gene variant using fixed- and random-effects models, in addition to the calculation of 95% confidence intervals (CI). Fixed-effects summary ORs were calculated using the Mantel-Haenszel method,[14], [15] and the DerSimonian and Laird method was used to calculate random-effects summary ORs [16]. The results were very similar for both summary OR so only one is presented. The frequency of at-risk genotypes was compared between cases and controls for each single nucleotide polymorphism (SNP) analyzed. For each meta-analysis, the I^2^ was calculated and a chi-squared test for heterogeneity was performed.[17] The genetic models used reflected those evaluated in the primary publication. For assessment of small-study bias, funnel plots and the Egger regression asymmetry test were conducted for each SNP with four or more publications.[18]

The proportion of haemorrhagic stroke cases in the population that could be attributed to a particular gene variant (population-attributable risk [PAR]) was estimated as follows:
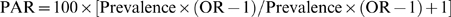
For this calculation the fixed effects model was used and the prevalence of exposure was estimated as the genotype frequency among control adults.

## Results

**Figure 1:**
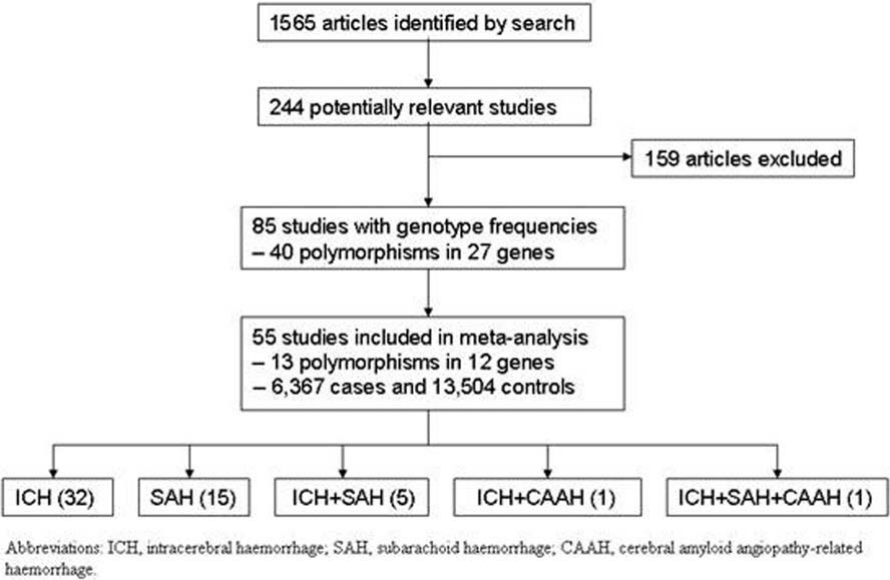
Flow chart illustrating number of studies included in the meta-analyses.

Our initial search identified 1,565 studies of which 85 met our inclusion criteria. In total, 40 polymorphisms in 27 genes were identified. Genes with three or more publications on each SNP were included in our analyses, leaving a total of 55 publications addressing 13 polymorphisms in 12 genes (Figure 1).

From the 12 genes analysed in detail (representing 6,367 cases and 13,504 controls), the mean number of studies per candidate gene was five. Six (46%) of the 13 meta-analyses had more than 500 cases, and 8 (62%) had a total participant size of greater than 1000 (Table 1). No single meta-analysis had more than 2000 cases.

**Table 1:** Details of size of studies included in each gene investigated.

Gene	Polymorphism	No. of studies	No. of cases	No. of controls	Average no. of cases/study	Largest study (cases/controls)	Smallest study (cases/controls)
**Apolipoprotein E**	ε2, ε3, ε4	11	1276	3531	125	333/634	48/24
**ACE**	I/D	8	744	1289	93	258/299	38/38
**SERPINA3**	A/T	6	1069	1294	178	437/405	38/80
**SERPINE1**	4G/5G	5	240	1233	48	60/485	31/60
**Factor XIII**	Val/Leu	5	531	1172	106	201/201	64/127
**MTHFR**	C677T	5	635	2275	127	503/1832	20/24
**eNOS**	T-786C	4	893	784	223	411/405	52/90
**Factor V**	Leiden R/Q 506 G/A	3	285	836	98	201/201	38/247
**Endoglin**	Exon7/8 G/A insertion	3	285	512	95	119/119	63/191
**Glycoprotein 1b-α**	VNTR repeat BCD	3	301	666	100	141/141	57/422
**Glycoprotein Ia**	Glu505Lys and C807T	3	286	590	95	141/141	42/346
**Glycoprotein IIIa**	Leu33Pro	3	286	590	95	141/141	42/346

Table 2 shows the genotypic odds ratios (ORs) for the 13 polymorphisms evaluated.

**Table 2:** Summary data of candidate genes in haemorrhagic stroke.

Gene	Polymorphism	Genetic model	Stroke subtype	Number of studies	Cases		Controls		OR (95% CI)	*P*het value
					Frequency of at risk allele	No.	Frequency of at risk allele	No.		
**Apolipoprotein E**	**ε2**, ε3, ε4	Dominant	All HS	11	0.174	1376	0.125	3531	1.17 (0.98, 1.41)	0.41
			ICH	9	0.072	929	0.053	3186	1.09 (0.88, 1.35)	0.30
			SAH	4	0.091	383	0.058	1877	1.20 (0.85, 1.70)	0.90
			CAAH	2	0.180	64	0.078	430	3.36 (1.71, 6.59)	0.38
			Lobar	4	-	244	-	393	1.81 (1.26, 2.62)	0.07
			Non-lobar	4	-	437	-	1115	1.08 (0.80, 1.47)	0.57
	ε2, ε3, **ε4**	Dominant	All HS	11	0.257	1376	0.231	3531	1.18 (1.01, 1.37)	0.36
			ICH	9	0.128	929	0.116	3186	1.17 (0.98, 1.40)	0.49
			SAH	4	0.120	383	0.112	1877	1.22 (0.90, 1.64)	0.15
			CAAH	2	0.180	64	0.176	430	2.69 (1.47, 4.92)	0.46
			Lobar	4	-	244	-	939	1.49 (1.08, 2.05)	0.36
			Non-lobar	4	-	437	-	1115	1.05 (0.81, 1.37)	0.41
**SERPINE1**	4G/**5G**	Recessive	All HS	5	0.490	240	0.443	1446	1.42 (1.03, 1.96)	0.99
			ICH	4	0.490	198	0.443	1102	1.40 (0.98, 2.00)	0.97
**ACE**	I/**D**	Recessive	All HS	8	0.487	744	0.544	1289	0.92 (0.75, 1.14)	0.12
			ICH	5	0.523	280	0.558	719	1.00 (0.73, 1.36)	0.11
			SAH	3	0.464	464	0.526	570	0.87 (0.65, 1.14)	0.49
	**I**/D	Recessive	All HS	8	0.513	744	0.456	1289	1.48 (1.20, 1.83)	0.02
			ICH	5	0.477	280	0.442	719	1.27 (0.90, 1.77)	0.20
			SAH	3	0.536	464	0.474	570	1.64 (1.24, 2.17)	0.009
**Factor V**	Leiden R/Q 506 G/**A**	Dominant	All HS	3	0.007	285	0.024	836	0.31 (0.11, 0.90)	0.69
			ICH	3	0.005	190	0.024	836	0.29 (0.08, 1.05)	0.50
			SAH	2	0.011	95	0.022	589	0.45 (0.11, 1.95)	0.61
**Endoglin**	Exon7/8 G/A insertion	Recessive	All HS	3	0.170	285	0.183	512	3.47 (1.45, 8.30)	0.56
**SERPINA3**	A/**T**	Recessive	All HS	6	0.493	1069	0.481	1294	1.10 (0.91, 1.33)	0.40
			ICH	4	0.553	452	0.431	626	1.28 (0.98, 1.67)	0.47
			SAH	2	0.450	616	0.453	668	0.94 (0.71, 1.24)	0.64
**eNOS**	T-786**C**	Dominant	SAH	4	0.127	893	0.133	784	1.27 (0.99, 1.62)	0.62
**MTHFR**	C677**T**	Recessive	ICH	5	0.449	635	0.42	2275	1.11 (0.89, 1.39)	0.12
**Factor XIII**	Val/**Leu**	Recessive	All HS	5	0.266	531	0.26	1172	1.36 (0.89, 2.08)	0.11
**Glycoprotein 1b-α**	VNTR repeat **B**CD	Dominant	All HS	3	0.075	301	0.041	666	1.04 (0.66, 1.64)	0.24
**Glycoprotein Ia**	Glu505**Lys**	Dominant	All HS	3	0.101	286	0.107	590	0.82 (0.56, 1.20)	0.36
	C807**T**	Dominant	All HS	3	0.386	286	0.369	590	1.15 (0.83, 1.59)	0.79
**Glycoprotein IIIa**	Leu33**Pro**	Dominant	All HS	3	0.133	286	0.164	590	0.76 (0.54, 1.07)	0.12

Apo ε2 (bold) vs others.

Apo ε4 (bold) vs others.

Abbreviations: OR, fixed effect odds ratio; P Het, P value for heterogeneity; ACE, gene encoding angiotensin converting enzyme; eNOS, gene encoding endothelial nitric oxide synthase; MTHFR, gene encoding methylenetetrahydrofolate reductase; I/D, insertion/deletion; VNTR, variable number tandem repeat; All HS, all haemorrhagic stroke; ICH, intracerebral haemorrhage; SAH, subarachnoid haemorrhage; CAAH, cerebral amyloid angiopathy-related haemorrhage.

Table shows both alleles with the one used for analysis in bold followed by the genetic model used for genotype characterisation. Apo ε2 (bold) vs others. Apo ε4 (bold) vs others.

### Apolipoprotein E:

**Figure 2:**
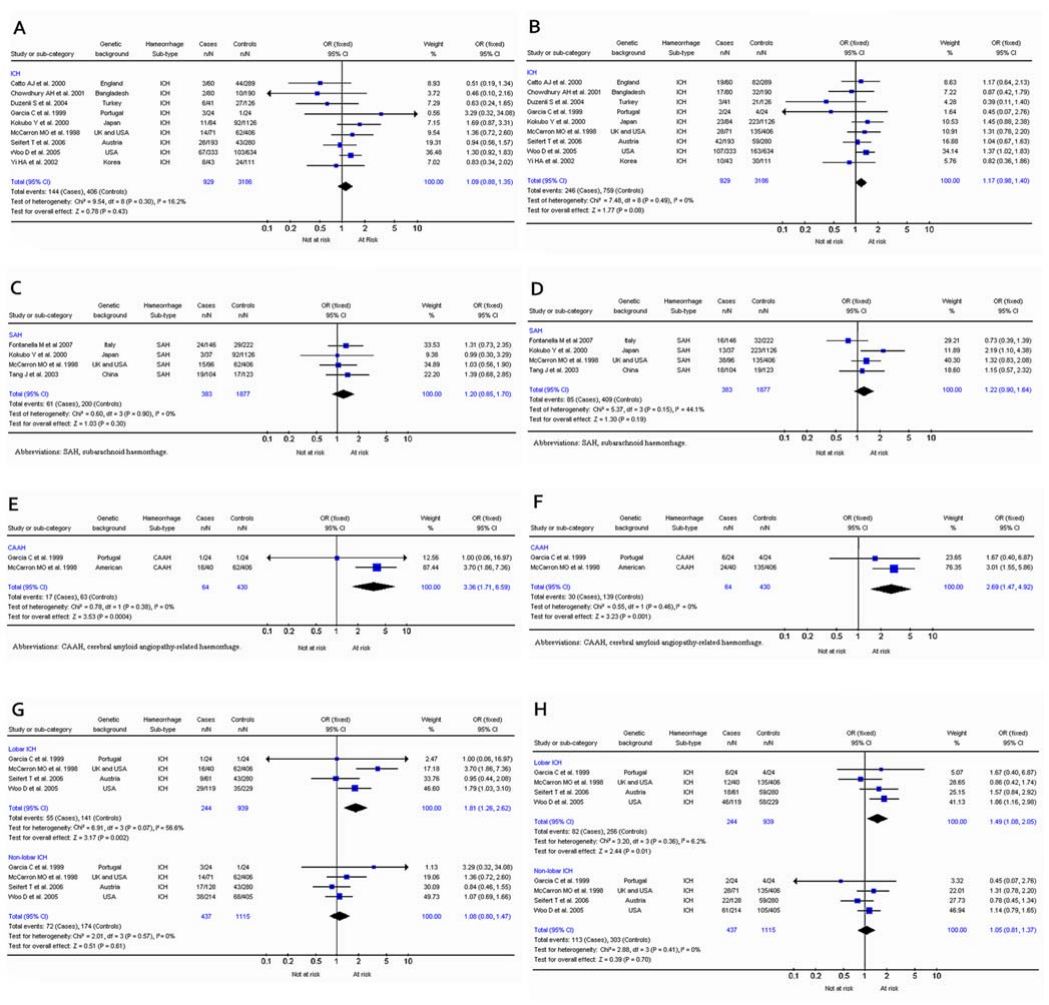
2a. Results for apolipoprotein &b.epsi;2 allele and intracerebral haemorrhage comparing carriers of the &b.epsi;2 allele with those with the &b.epsi;3 and &b.epsi;4 alleles. 2b. Results for apolipoprotein &b.epsi;4 allele and intracerebral haemorrhage comparing carriers of the &b.epsi;4 allele with those with the &b.epsi;2 and &b.epsi;3 alleles. 2c. Results for apolipoprotein &b.epsi;2 allele and subarachnoid haemorrhage comparing carriers of the &b.epsi;2 allele with those with the &b.epsi;3 and &b.epsi;4 alleles. 2d. Results for apolipoprotein &b.epsi;4 allele and subarachnoid haemorrhage comparing carriers of the &b.epsi;4 allele with those with the &b.epsi;2 and &b.epsi;3 alleles. 2e. Results for apolipoprotein &b.epsi;2 allele and cerebral amyloid angiopathy-related haemorrhage comparing carriers of the &b.epsi;2 allele with those with the &b.epsi;3 and &b.epsi;4 alleles. 2f. Results for apolipoprotein &b.epsi;4 allele and cerebral amyloid angiopathy-related haemorrhage comparing carriers of the &b.epsi;4 allele with those with the &b.epsi;2 and &b.epsi;3 alleles. 2g. Results for apolipoprotein &b.epsi;2 allele and lobar or non-lobar haemorrhage comparing carriers of the &b.epsi;2 allele with those with the &b.epsi;3 and &b.epsi;4 alleles. 2h. *Results* for apolipoprotein &b.epsi;4 allele and lobar or non-lobar haemorrhage comparing carriers of the &b.epsi;4 allele with those with the &b.epsi;2 and &b.epsi;3 alleles.

The most investigated gene was apolipoprotein E, with 11 studies [19]–[29] that included 1376 cases and 3531 controls. APOE ε2, ε3 and ε4 alleles were analysed in a dominant model (i.e. ε2ε4+ε3ε4+ε4ε4 vs all other genotypes or ε2ε2+ε2ε3+ε2ε4 vs all other genotypes). For intracerebral haemorrhage, no association was found for carriers of the ε2 allele (Figure 2a) (OR, 1.09; 95% CI, 0.88–1.35), but an association was observed for carriers of the ε4 allele (Figure 2b) with an OR of 1.17 (95% CI, 0.98–1.40; P = 0.08). No significant interstudy OR heterogeneity was observed for either allele (χ^2^ = 9.54; *P*_Het_ = 0.30 and χ^2^ = 7.48; *P*_Het_ = 0.49 respectively).

For subarachnoid haemorrhage, no association was found for carriers of the ε2 (Figure 2c) or ε4 (Figure 2d) allele with ORs of 1.20 (95% CI, 0.85–1.70; p = 0.30) and 1.22 (95% CI, 0.90–1.64; p = 0.19), respectively. No significant interstudy OR heterogeneity was observed for either allele.

Only two publications (64 cases and 430 controls) with genotype frequencies addressed cerebral amyloid angiopathy-related haemorrhage. McCarron et al. [23] used histology to confirm a diagnosis of CAAH, while Garcia et al. [25] used neuroimaging and clinical criteria to diagnose ‘probable/possible’ CAAH. Carriers of the ε2 allele demonstrated an OR of 3.36 (95% CI, 1.71–6.59; p = 0.0004) (Figure 2e) and for carriers of ε4 an OR of 2.69 (95% CI, 1.47–4.92; p = 0.001) (Figure 2f) for this condition. No significant interstudy OR heterogeneity was observed for either allele (ε2: χ^2^ = 0.78; *P*_Het_ = 0.36 and ε4: χ^2^ = 0.55; *P*_Het_ = 0.45).

Previous studies investigating apolipoprotein E in haemorrhagic stroke have addressed the association of the ε2 and ε4 alleles in lobar and non-lobar haemorrhage.[30] Four studies [20], [22], [23], [25] provided data that appropriately categorized haemorrhage location as lobar or non-lobar. Both ε2 and ε4 alleles were significantly associated with lobar haemorrhage (OR, 1.81; 95% CI, 1.26–2.62; P = 0.002 and OR, 1.49; 95% 1.08–2.05; P = 0.01 respectively) but not with non-lobar haemorrhage (OR, 1.08; 95% CI, 0.80–1.47 and OR, 1.05; 95% CI, 0.81–1.37) (Figures 2g and 2h). No significant interstudy OR heterogeneity was observed for either allele (ε2 lobar: χ^2^ = 6.91; *P*_Het_ = 0.07; ε2 non-lobar: χ^2^ = 2.01; *P*_Het_ = 0.57; ε4 lobar: χ^2^ = 3.20; *P*_Het_ = 0.36; ε4 non-lobar: χ^2^ = 2.88; *P*_Het_ = 0.41).

### SERPINE1:

**Figure 3:**
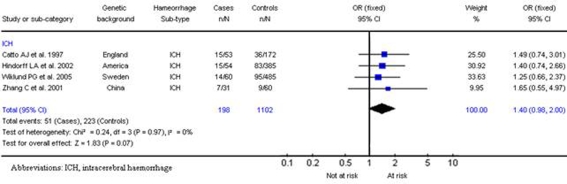
Results for SERPINE1 4G/5G and intracerebral haemorrhage of *5G* allele homozygous (5G/5G) genotype.

A total of five studies [31]–[35] (240 cases and 1233controls) investigated the SERPINE1 (also known as plasminogen activator inhibitor-1) 4G/5G polymorphism. Four addressed intracerebral haemorrhage and one study [33] addressed intracerebral haemorrhage, subarachnoid haemorrhage and ruptured intracranial aneurysm. For intracerebral haemorrhage alone an association was observed for those homozygous for the *5G* allele with an OR of 1.40 (95% CI, 0.98–2.00; *P* = 0.07) (Figure 3). When subarachnoid haemorrhage and ruptured intracranial aneurysm cases were included in this analysis a significant association was observed with a similar OR of 1.42 (95% CI, 1.03–1.96; *P* = 0.03). No significant interstudy OR heterogeneity was observed (χ^2^ = 0.28; *P*_Het_ = 0.99). The distribution of the OR in relation to its standard deviation in the funnel plot was symmetrical, and the Egger test result was not significant (*P* = 0.178), suggesting a low probability of small-study bias.

### Angiotensin-Converting Enzyme:

A total of 8 studies [36]–[43] (744 cases and 1289 controls) were identified that evaluated the ACE/ID polymorphism of which five related to intracerebral haemorrhage, one for subarachnoid haemorrhage and two for ruptured intracranial aneurysm.

**Figure 4:**
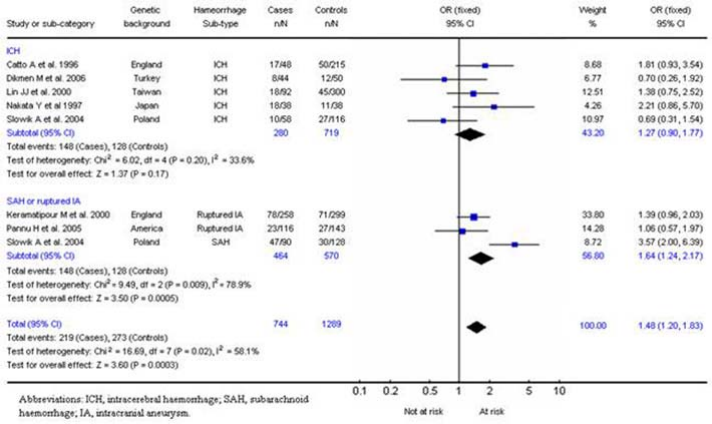
Results for ACE/II gene and haemorrhagic stroke.

No significant association for haemorrhagic stroke was found for those homozygous for the *D* allele (ACE/DD) for intracerebral haemorrhage alone (OR, 1.00; 95% CI, 0.73–1.36; p = 1.00), or with subarachnoid haemorrhage or ruptured intracranial aneurysm (OR, 0.87; 95% CI, 0.65–1.14; p = 0.09). In addition to examining the association of the ACE/DD genotype with haemorrhagic stroke, several studies have suggested that the ACE/II genotype may be associated with haemorrhagic stroke. When all studies were evaluated together, those homozygous for the ACE/I allele demonstrated an overall pooled OR of 1.48 (95% CI, 1.20–1.83; p = 0.0003) (Figure 4). Significant interstudy OR heterogeneity was observed (χ^2^ = 16.69; *P*_Het_ = 0.02), however a random-effect model that takes into account the intra- and inter-study variability resulted in a similar overall estimate that remained significant (OR, 1.45; 95% CI, 1.01–2.07; p = 0.04). The distribution of the OR in relation to its standard deviation in the funnel plot was symmetrical, and the Egger test result was not significant (p = 0.667), suggesting a low probability of small-study bias. For intracerebral haemorrhage alone this association did not remain significant (OR, 1.27; 95% CI, 0.90–1.77; *P* = 0.17). However, for subarachnoid haemorrhage or ruptured intracranial aneurysm, a summary OR, under a fixed-effect model, of 1.64 (95% CI, 1.24–2.17; p = 0.0003) was observed.

### Factor V:

**Figure 5:**
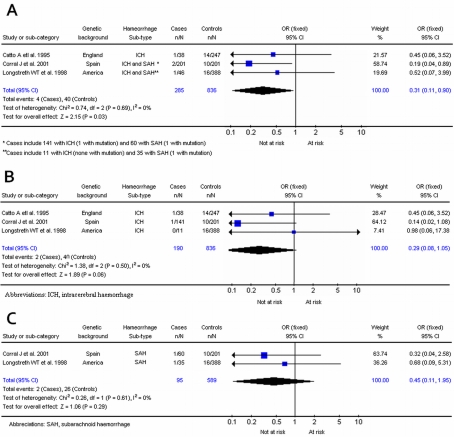
5a. Results for Factor V Leiden mutation and haemorrhagic stroke. 5b. Results for Factor V Leiden mutation and ICH. 5c. Results for Factor V Leiden mutation and SAH.

Three studies (285 cases, 836 controls) investigated haemorrhagic stroke in the factor V Leiden mutation. One addressed intracerebral haemorrhage [44] and two addressed both intracerebral haemorrhage and subarachnoid haemorrhage [45], [46]. When considered in totality, carriers of the *adenine* allele were less likely to develop haemorrhagic stroke (OR, 0.31; 95% CI, 0.11–0.90; p = 0.03) when compared to wild-type (*guanine/guanine*) (Figure 5a). No significant interstudy heterogeneity was observed (χ^2^ = 0.74; *P*_Het_ = 0.69)

For intracerebral haemorrhage alone, a summary OR, under the fixed-effects model, of 0.29 (95% CI, 0.08–1.05; p = 0.06) was observed for adenine allele homozygotes (Figure 5b). For subarachnoid haemorrhage alone, a summary OR, under a fixed-effect model, of 0.45 (95% CI, 0.11–1.95; p = 0.29) was observed (Figure 5c). No significant interstudy heterogeneity was observed for intracerebral (χ^2^ = 1.38; *P*_Het_ = 0.50) or subarachnoid haemorrhage (χ^2^ = 0.26; *P*_Het_ = 0.61).

### Endoglin:

Three studies (285 cases; 512 controls) investigated risk of haemorrhagic stroke and the exon 7/8 GA insertion of the gene encoding endoglin. One study addressed intracerebral haemorrhage [47] and two studies addressed subarachnoid haemorrhage [48], [49]. When considered in totality, those homozygous for the *insertion* allele demonstrated an OR of 3.47 (95% CI, 1.45–8.30; *p* = 0.005). No significant interstudy OR heterogeneity was observed (χ^2^ = 1.15; *P*_Het_ = 0.56).

### SERPINA3:

A total of 6 studies [50]–[55] (1069 cases and 1294 controls) were identified that evaluated the polymorphism in the gene encoding SERPINA3 (also known as α1-antichymotrypsin) where alanine is replaced by threonine. Four of these addressed intracerebral haemorrhage and two addressed subarachnoid haemorrhage.

**Figure 6:**
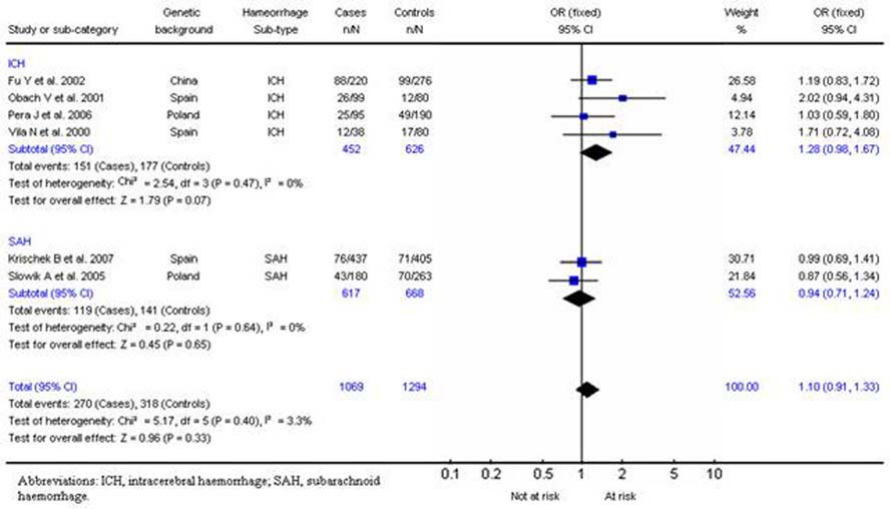
Results for SERPINA3/TT in haemorrhagic stroke.

For intracerebral haemorrhage, a summary OR, under the fixed-effects model, of 1.28 (95% CI, 0.98–1.67; p = 0.07) was observed for individuals homozygous for the T allele compared with A allele carriers (AT+AA). For subarachnoid haemorrhage alone, a summary OR, under a fixed-effect model, of 0.94 (95% CI, 0.71–1.24; p = 0.65) was observed (Figure 6). No significant interstudy heterogeneity was observed (χ^2^ = 5.17; *P*_Het_ = 0.40) and the funnel plot distribution was symmetrical with an insignificant Egger test (*P* = 0.14), indicating a low probability of small-study bias.

### Endothelial nitric oxide synthase:

**Figure 7:**
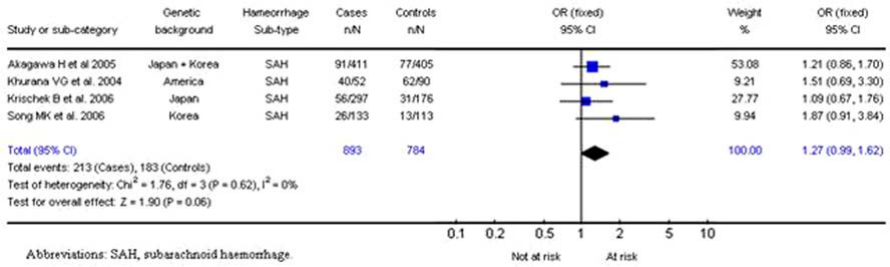
Results for eNOS/786C polymorphism and subarachnoid haemorrhage.

The eNOS *T*786*C* mutation was evaluated in 4 studies [56]–[59], all of which addressed subarachnoid haemorrhage with a total of 893 cases and 784 controls. The summary OR under a fixed-effect model showed an OR for dominant carriers of the 786C mutation of 1.27 (95% CI, 0.99–1.62; *P* = 0.06) (Figure 7). No significant interstudy heterogeneity was observed (χ^2^ = 1.76; *P*_Het_ = 0.62). The distribution of the ORs from individual studies in relation to their respective standard deviations was symmetrical, and the Egger test result suggested a low probability of small-study bias (p = 0.29).

### Methylenetetrahydrofolate reductase:

**Figure 8:**
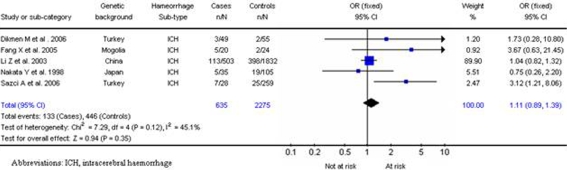
Results for MTHFR/677TT gene and intracerebral haemorrhage.

Five studies [60]–[64] investigated the MTHFR C677T polymorphism, all addressing intracerebral haemorrhage (635 cases; 2275 controls). No significant association for intracerebral haemorrhage was found for ^677^TT (OR, 1.11; 95% CI, 0.89–1.39; p = 0.35) (Figure 8). No significant interstudy heterogeneity was observed (χ^2^ = 7.29; *P*_Het_ = 0.12) and the funnel plot distribution was symmetrical with an insignificant Egger test (p = 0.27), indicating a low probability of small-study bias.

### Factor XIII:

The factor XIII valine/leucine polymorphism was investigated in 5 studies [33], [65]–[68]. Subarachnoid haemorrhage was addressed in addition to intracerebral haemorrhage in two of these studies (531 cases; 1172 controls). When considered in totality, those homozygous for the *leucine* allele demonstrated an OR of 1.39 for haemorrhagic stroke (95% CI, 0.89–2.08), although this result was not statistically significant (p = 0.15). No significant interstudy heterogeneity was observed (χ^2^ = 7.45; *P*_Het_ = 0.11) and the funnel plot distribution was symmetrical with an nonsignificant Egger test (p = 0.27), indicating a low probability of small-study bias. For intracerebral haemorrhage, a summary OR under a fixed-effect model of 1.32 (95% CI, 0.82–2.06; p = 0.27) was observed for individuals homozygous for the *leucine* allele compared with *valine* allele carriers (*V/L* plus *V/V*)).

### Glycoprotein Ib-α:

Three studies investigated haemorrhagic stroke in the glycoprotein 1b-α VNTR polymorphism. Two addressed intracerebral haemorrhage [69], [70] (198 cases; 563 controls) and one addressed subarachnoid haemorrhage [71] (103 cases; 103 controls). When considered in totality carriers of the B group of tandem repeats had no associated risk for haemorrhagic stroke with a summary OR, under a fixed-effect model, of 1.04 (95% CI, 0.66–1.64; p = 0.85).

### Glycoprotein Ia and IIIa:

Three studies investigated haemorrhagic stroke in the glycoprotein Ia Glu505Lys polymorphism [69], [71], [72] (286 cases; 590 controls). No association for haemorrhagic stroke was found for carriers of the lysine allele with a summary OR, under the fixed-effect model, of 0.82 (95% CI, 0.56–1.20). Additionally, the same studies found no association for haemorrhagic stroke with the C807T polymorphism of glycoprotein Ia (OR, 1.15; 95% CI, 0.83–1.59) nor for the glycoprotein IIIa Leu33Pro polymorphism (OR, 0.76; 95% CI, 0.54–1.07).

## Discussion

In this comprehensive meta-analysis, four of the 13 (31%) polymorphisms analyzed significantly increased the risk of haemorrhagic stroke. The mean number of participants in two of these meta-analyses (ACE and SERPINE1) was more than 1850, allowing more precise estimates to be made of the effect of these genes than from any single study. The individual risk provided by these candidate genes was moderate (OR, 1.48; 95% CI: 1.20–1.83 and 1.42, 95% CI: 1.03–1.96, respectively). This effect size is in broad agreement with previous studies in ischaemic stroke [6] and other complex diseases that are thought to have a polygenic basis, such as ischaemic heart disease [73], [74]. Further, our study suggests that the hypercoagulable state conferred by the factor V Leiden mutation is protective against haemorrhagic stroke (OR, 0.31; 95% CI, 0.11–0.90; p = 0.03). This is in accordance with previous studies that have suggested a protective role for this polymorphism in other bleeding disorders. [75], [76] However, these results are based on a smaller dataset compared to ischaemic stroke making the point estimates less reliable. The seven gene variants in the six remaining genes – factor XIII, MTHFR, SERPINA3 and glycoproteins Ia, 1b-α, and IIIa – have so far failed to provide evidence to support an increased susceptibility to haemorrhagic stroke.

The majority of candidate genes in haemorrhagic stroke have been investigated initially for their potential role in ischaemic stroke. For APOE, our results are in accordance with previous histological studies suggesting that, while the ε4 allele enhances the deposition of amyloid-β in peripheral (lobar) cerebral vasculature, the ε2 allele predisposes to the rupture of these lobar amyloid-β laden vessels, possibly through the promotion of fibrinoid necrosis [77]–[79]

A meta-analysis of genetic studies in ischaemic stroke by our group in 2004 investigated, among others, 9 (75%) of the 12 candidate genes in our study (Table 3) and involved approximately 3 times as many cases and controls. Although ischaemic and haemorrhagic stroke may share some common risk factors they are thought to have quite distinct molecular pathogeneses. Our current work demonstrates strikingly similar genetic ORs between the two major pathologies supporting a similar aetiological role for both ischaemic and haemorrhagic stroke (Table 3) with a greater patho-aetiological similarity existing between small vessel ischaemic disease and deep cerebral haemorrhage. Interestingly, Factor V was associated with ischaemic stroke and protective for haemorrhagic stroke, providing a biological relevant and important role for its aetiology in cerebrovascular disease.

**Table 3:** Odds ratios and 95% CIs of gene variants investigated in both ischaemic and haemorrhagic stroke. Data taken from Casas et al.^104^ and Ariyaratnam et al.^97^

Gene	ACE	ACE	Factor V	PAI-1	MTHFR	APOE	APOE
Polymorphism	I/D	I/D	Arg506Gln	4G/5G	C677T	e2/e3/e4	e2/e3/e4
Stroke Subtype							
**Caucasian Ischaemic stroke**	1.21 (1.08–1.35)	-	1.33 (1.12–1.58)	1.47 (1.13–1.92)	1.24 (1.08–1.42)	-	0.96 (0.84–1.11)
**Non-European Ischaemic Stroke**	1.82 (1.28–2.60)	-	-	-	1.22 (0.98–1.52)	-	1.77 (1.30–2.39)
**Haemorrhagic stroke**	0.92 (0.75, 1.14)	1.48 (1.20, 1.83)	0.30 (0.10, 0.87)	1.42 (1.03–1.96)	1.11 (0.89, 1.39)	1.17 (0.98–1.41)	1.18 (1.01–1.37)
**Intracerebral haemorrhage**	1.00 (0.73–1.36)	1.27 (0.90–1.77)	-	1.40 (0.98–2.00)	1.11 (0.89, 1.39)	1.09 (0.88–1.35)	1.17 (0.98–1.40)
**Subarachnoid haemorrhage**	0.87 (0.65–1.14)	1.64 (1.24–2.17)	-	-	-	1.20 (0.85–1.70)	1.22 (0.90–1.64)
**Cerebral amyloid angiopathy**	-	-	-	-	-	3.36 (1.71–6.59)	2.69 (1.47–4.92)

The population-attributable risks (PARs) for gene variants with the most reliable associations for haemorrhagic stroke in this study were 9.24% for the ACE/ID polymorphism and 7.78% for the SERPINE1 *4G/5G*. These values are lower than those reported for well-established risk factors for haemorrhagic stroke, such as hypertension [80] but not surprising, because the genetic contribution of any single gene toward a complex disease is unlikely to act in a simple mendelian fashion but rather with epistatic (gene-gene or gene-environmental interaction) effects. Nevertheless, given the high incidence of haemorrhagic stroke (100,000 per year in the United States), if these estimates hold true they suggest that common variants in the ACE and SERPINE1 genes alone may contribute between 8,000–10,000 haemorrhagic strokes in the United States each year.

The interpretation of any meta-analysis must be made within the context of its limitations, including study selection, publication bias, and variability in the methodological quality of the included studies. A comprehensive search was undertaken for all relevant studies including non-English language [19], [55], [81]. Although publication bias can never be completely excluded, many of the individual studies included in our meta-analyses were not statistically significant and were interpreted by their authors as negative studies suggesting a desire by authors to submit, and a willingness by editors to publish such work. In addition, Egger asymmetry tests and funnel plots showed no substantial evidence of publication bias in the nine meta-analyses for which four or more studies had been published (apolipoprotein E *ε*4, and *ε*2; SERPINA3 *A/T*; eNOS *T786C*; ACE/ID; MTHFR *C677T*; factor XIII *Val/Leu* and SERPINE1 *4G/5G*), although it is acknowledged that at times the study numbers for each gene were small as was the number of subjects for each study making the point estimates less reliable when compared against our previous larger meta-analyses in stroke [5], [6]. Moreover, rigorous selection criteria (use of neuroimaging or autopsy to diagnose haemorrhagic stroke) enriched the meta-analyses for studies with comparable selection of participants and haemorrhagic stroke sub-types. Notwithstanding these selection criteria, patients with severe ICH from any aetiology who died at onset without gaining access to secondary care could not have entered in any of the included manuscripts.

Only two of our meta-analyses included more than 1000 cases (apolipoprotein E and SERPINA3). Reliable interpretation of the association of candidate genes with haemorrhagic stroke will only come from studies with an order of magnitude larger than those performed to date. Dichgans et al.[82] suggest that to confirm odds ratios between 1.2–1.5 in candidate genes with allele frequencies between 0.1–0.5, sample sizes of 800–20,000 are required to achieve reliable statistical significance at a *P* value of at least 0.05. The average number of cases and controls in our meta-analysis were just 578 and 1224 respectively. Achieving appropriate sample sizes is only likely to be achieved using a multicenter collaborative approach, with studies using uniform criteria for the selection of cases and controls and submitting both positive and negative data to a common database for continuously updated and cumulative meta-analysis. Using neuroimaging- or autopsy-diagnosed haemorrhagic stroke as a selection criteria may have helped to maintain comparable groups of cases. However the selection of control groups varied considerably between studies with some excluding control subjects with established hypertension, while others argued that as not all patients with haemorrhagic stroke have high blood pressure such exclusion of control population is unnecessary. Statistical methods using marker genotype data may in the future permit the detection and control of confounding due to population stratification and selection bias in genetic association studies.[83] This may reduce the impact of variability within controls groups, although the strategy of using a control group that has not been specifically phenotyped for comparison with each individual disease has recently been successfully employed [84] suggesting that this is not a major problem.

All studies from different ancestral backgrounds were included. Although there is evidence to suggest that allele frequencies for several candidate genes investigated in our study vary between ethnicity, and epidemiological studies suggest differences in the incidence and prevalence of haemorrhagic stroke in certain ethnic groups with the rate in black Americans twice that of white Americans,[85], [86] as well as a greater incidence in Japanese population when compared to Caucasian populations [87], the majority of our studies were conducted in Caucasians. Indeed, we have recently shown in ischaemic stroke that differences in genetic effects across different ethnic groups may be overstated [5]. However, the effect of heterogeneity by ethnicity cannot be completely dismissed.

Only adults with ruptured intracranial aneurysm were included in our analyses addressing subarachnoid haemorrhage. Many published articles have investigated genetic polymorphisms in subjects with unruptured intracranial aneurysm. While some genes may predispose to the formation of intracranial aneurysms, others may predispose to rupture of aneurysms. By performing a similar meta-analysis of genetic association studies in unruptured intracranial aneurysm it may be possible to further our understanding of the genetic and molecular pathogenesis of subarachnoid haemorrhage. Although attempts were made to analyse sub-types of haemorrhage separately, the majority of the time such data were not readily available. For this, as well as reasons of aetiology where it can be argued that haemorrhagic stroke at its most basic consists of vessel rupture, data were combined. Readers should therefore assess this manuscript with this limitation in mind. Further, even within the category of primary intracerebral haemorrhage, heterogeneity may exist while some patients may not have been subjected to detailed investigations in order to determine secondary underlying causes, thus erroneously labelling them clinically as ‘primary’.

Although it is not possible to exclude the future identification of one or more genes with a more substantial effect on risk of haemorrhagic stroke using different models such as genome wide association searches, our findings based upon candidate gene strategies suggest that several genes, each with a small to moderate effect, are likely to act individually, together, or in combination with environmental determinants to contribute towards haemorrhagic stroke. One implication of these findings is that predictive genetic tests that use any single variant are unlikely individually to have much value. However, tests that combine genotyping for one or more risk alleles and that integrate the results with established risk prediction tools based on acquired risk factors may have greater utility.[88], [89]

This study may have important implications for those involved in exploring the genetic aetiology of blood pressure because of the relationship between haemorrhagic stroke and hypertension. Few studies (∼50%) reported the prevalence of hypertension among their case and control populations. Of those that did the average incidence of hypertension among cases in the ACE study was 51.7% compared to 29.7% in controls, and for PAI-1 the average for cases was 26.7% and for controls 12.9%. From our study the mechanism by which causative SNPs lead to haemorrhagic stroke cannot be certain, although this could be via known causal pathways such as hypertension especially if those genes are involved in blood pressure regulation. There is, therefore, a clear need for future investigators and editors to ensure that this important variable is documented and published in the final manuscripts.

In summary, this study suggests the existence of a genetic basis for some types of haemorrhagic stroke with no single gene but rather demonstrates a polygenic aetiology. However, the evidence base is smaller than when compared against ischaemic stroke, although the odds ratios are of similar magnitude. An international collaborative approach is more likely to lead to a sufficient number of subjects being recruited for the reliable identification of risk alleles in haemorrhagic stroke.
